# Insights Into the Properties, Biological Functions, and Regulation of USP21

**DOI:** 10.3389/fphar.2022.944089

**Published:** 2022-06-30

**Authors:** Tao An, Yanting Lu, Xu Yan, Jingjing Hou

**Affiliations:** ^1^ School of Pharmaceutical Sciences, Qilu University of Technology (Shandong Academy of Sciences), Jinan, China; ^2^ College of TCM, Shandong University of Traditional Chinese Medicine, Jinan, China; ^3^ Department of Gastrointestinal Surgery, School of Medicine, Institute of Gastrointestinal Oncology, Zhongshan Hospital of Xiamen University, Xiamen University, Xiamen, China

**Keywords:** USP21, deubiquitylation, signaling pathways, regulation, cancer, target inhibition

## Abstract

Deubiquitylating enzymes (DUBs) antagonize ubiquitination by removing ubiquitin from their substrates. The role of DUBs in controlling various physiological and pathological processes has been extensively studied, and some members of DUBs have been identified as potential therapeutic targets in diseases ranging from tumors to neurodegeneration. Ubiquitin-specific protease 21 (USP21) is a member of the ubiquitin-specific protease family, the largest subfamily of DUBs. Although USP21 was discovered late and early research progress was slow, numerous studies in the last decade have gradually revealed the importance of USP21 in a wide variety of biological processes. In particular, the pro-carcinogenic effect of USP21 has been well elucidated in the last 2 years. In the present review, we provide a comprehensive overview of the current knowledge on USP21, including its properties, biological functions, pathophysiological roles, and cellular regulation. Limited pharmacological interventions for USP21 have also been introduced, highlighting the importance of developing novel and specific inhibitors targeting USP21.

## Introduction

Ubiquitin (Ub) is a 76-amino acid protein modifier that can be covalently conjugated to lysine residues in target proteins through a cascade known as ubiquitylation or ubiquitination (Komander and Rape, 2012). Briefly, Ub is initially activated by Ub-activating enzyme E1, followed by conjugation to Ub-conjugating enzyme E2 and substrate targeting by E3 Ub ligase (Yang et al., 2009). The consequences of ubiquitylation vary with the patterns of ubiquitylation (monoubiquitylation versus polyubiquitylation), linkage types and lengths of the Ub chain, which are essential for the regulation of protein stability, activity, molecular interactions, and subcellular localization (Komander and Rape, 2012; Kwon and Ciechanover, 2017). Therefore, ubiquitylation, a pivotal post-translational modification, regulates virtually all cellular processes, and defects in this process play a major role in distinct diseases (Popovic et al., 2014).

Conversely, ubiquitylation can be reversed by deubiquitylating enzymes (DUBs), which remove Ub moieties from substrates (Mevissen and Komander, 2017). In humans, there are approximately 100 DUBs, which are classified into seven families depending on their sequence and domain conservation: ubiquitin-specific proteases (USPs), ubiquitin carboxy-terminal hydrolases (UCHs), ovarian tumor proteases (OTUs), Machado-Joseph disease proteases (MJDs), MIU-containing novel DUB family (MINDY) proteases, Jab1/MPN/MOV34 metalloenzymes (JAMMs), and Zn-finger and UFSP domain proteins (ZUFSPs). All of these are cysteine peptidases, except for JAMMs (Mevissen and Komander, 2017; Hermanns et al., 2018). Since protein ubiquitylation is controlled by the coordinated activity of Ub E3 ligases and DUBs, it is not difficult to imagine that DUBs are also significant regulators of many physiological and pathological processes. Dysfunction of numerous DUBs, such as USP7 and OTUB1, has been implicated in tumors and other pathologies, including neurodegenerative, immune, and infectious diseases (Harrigan et al., 2018; Nininahazwe et al., 2021; Liao et al., 2022). Accordingly, DUBs have attracted attention as interesting therapeutic targets, and a large number of DUB inhibitors have been developed, with some now moving towards or into clinical assessment (Harrigan et al., 2018).

**FIGURE 1:**
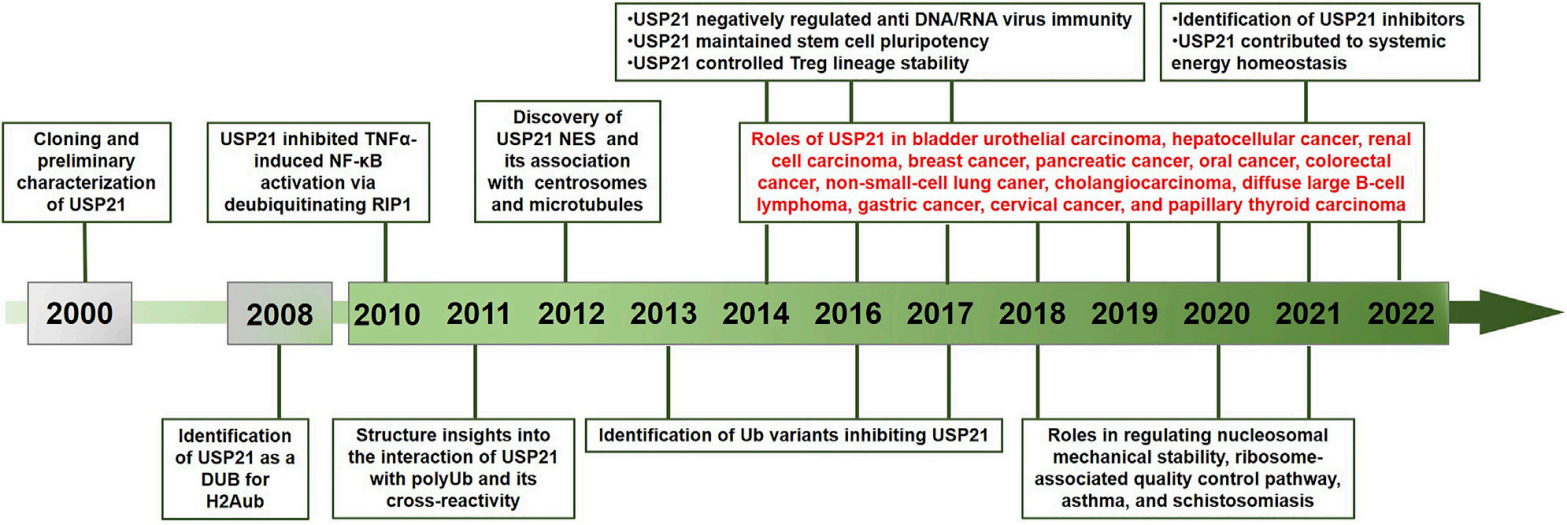
Timeline of USP21 research: selected discoveries are listed, and antitumor effects are marked in red. DUB, deubiquitylating enzyme; RIP1, receptor interacting protein 1; NES, nuclear export sequence.

Ubiquitin-specific protease 21 (USP21), also designated USP23 by Smith et al., is a cysteine DUB belonging to the USP family (Smith and Southan, 2000). In 2000, both a truncated USP21 form with an N-terminal deletion and full-length USP21 were separately discovered, cloned, and characterized by two groups (Gong et al., 2000; Smith and Southan, 2000). Monoubiquitylated histone H2A, as the first substrate of USP21, was only identified in 2008 (Nakagawa et al., 2008), and a large body of knowledge about USP21 has emerged since 2010 (Figure 1). USP21 is now known to deubiquitinate multiple well-known target proteins related to crucial processes involved in both cellular homeostasis and disease, especially cancer, where USP21 is frequently dysregulated.

In this review, we sought to present a comprehensive description of current knowledge concerning all aspects of USP21. First, we summarize the properties of USP21, including its expression, localization, structure, and catalytic activity. Second, we discuss the cellular functions of USP21 and its pathological roles in diseases, with a summary of its protein substrates (Tables 1–3). Third, we document several mechanisms involved in the cellular regulation of USP21. Finally, we address the developmental status of pharmacological interventions of USP21 (Tables 4, 5). Taken together, the present review aimed to provide fundamental insights into the potential of USP21 as a therapeutic target in diverse diseases, including cancer.

**TABLE 1:** Substrates of USP21 in signaling pathways.

Substrate	Type of ubiquitination removed by USP21	Results	References
NF-κB signaling			
RIP1	K63-linked polyubiquitination	Downregulation of TNFα-induced NF-κB activation	Xu et al. (2010)
IL-33	None reported	Protein stabilization promoting the transcription of p65	Tao et al. (2014)
Hippo signaling			
MARKs	None reported	Protein stabilization with resultant suppression of YAP/TAZ	Nguyen et al. (2017)
FoxM1	None reported	FOXM1 stabilization contributed to the nuclear translocation of YAP	Li et al. (2022)
MAPK/ERK signaling			
MEK2	K48-linked polyubiquitination	Protein stabilization activating ERK signaling	Li et al. (2018)
GATA3	None reported	Protein stabilization stimulating the expression of MAPK1	Guo Q. et al. (2021)
Wnt signaling			
TCF7	None reported	Protein stabilization leading to activation of Wnt signaling	Hou et al. (2019)
Hedgehog signaling			
Gli1	None reported	Gli1-dependent transcription was suppressed by USP21 depletion or overexpression	Heride et al. (2016)
KCTD6	None reported	Unclear	Heride et al. (2016)

**TABLE 2:** Substrates of USP21 in biological processes.

Substrate	Type of ubiquitination removed by USP21	Results	References
Epigenetic regulation			
H2Aub	Monoubiquitylation	Decrease in the levels of H2Aub with concomitant regulation effect on other histone modifications	Nakagawa et al. (2008), Okuda et al. (2013), Bhattacharya et al. (2016), Jin et al. (2016), Peng et al. (2016a), Jullien et al. (2017), Xiao et al. (2020)
EZH2	None reported	Unknown	Chen et al. (2017b), Ma et al. (2021)
Centrosome and microtubule-associated functions			
Microtubules and centrosomes	None reported	Promoted the regeneration of microtubule network, the formation of primary cilium, and neurite outgrowth	Urbe et al. (2012)
Microtubule-associated proteins other than MARKs	None reported	Unknown	Urbe et al. (2012)
MARK3	None reported	Essential for macropinocytosis	Hou et al. (2021)
DNA repair			
BRCA2	None reported	Protein stabilization elevating homologous recombination efficiency	Liu et al. (2017)
Antiviral response and immune regulation			
RIG-I	K63-linked polyubiquitination	USP21 negatively regulated immune responses to RNA virus infection	Fan et al. (2014)
STING	K27/63 linked polyubiquitination	USP21 negatively regulated anti–DNA virus immunity	Chen et al. (2017a), Wu et al. (2021)
Tat	K48/63 linked polyubiquitination	USP21 inhibited HIV-1 replication	Gao et al. (2021)
GATA3	K48-linked polyubiquitination	Protein stabilization crucial for the physiological function of Treg cells	Zhang et al. (2013), Guo Q. et al. (2021)
FOXP3	None reported	Protein stabilization essential for Treg lineage stability *in vivo*	Li et al. (2016)
AIM2	K48-linked polyubiquitination	Protein stabilization promoting AIM2 inflammasome activation upon DNA stimulation	Hong et al. (2021)
PD-L1	None reported	Protein stabilization contributed to inhibit the function of effector T cells, and hence allow cancer cells escape immunity attack	Yang et al. (2021)
Embryonic stem cell maintenance and X chromosome inactivation			
Nanog	K48-linked polyubiquitination	USP21 maintains the stemness of mouse embryonic stem cells and may cause the disruption of XCI in androgenetic CHM	Liu et al. (2016), Jin et al. (2016), Kwon et al. (2017), Chen X. et al. (2021)
Other functions			
Tip5	None reported	Protein stabilization regulating rRNA gene transcription	Khan et al. (2015)
Es10 and Us10	Monoubiquitylation	Enhanced readthrough of ribosome stalls	Garshott et al. (2020)
Goosecoid	Monoubiquitylation	Craniofacial development	Liu et al. (2019)
DSCAM and DSCAML1	None reported	None reported	Sachse et al. (2019)
MLKL	None reported	Necroptosis regulation	Liu et al. (2021)

**TABLE 3:** Multifaceted role of USP21 in diseases.

Diseases	Substrates	Biological functions	References
Cancer			
Hepatocellular carcinoma	H2Aub	H2Aub downregulation, which was associated with increase in the mitotic mark H3S10p and the expression of oncogenic lipocalin 2	Bhattacharya et al. (2016)
	BRCA2	USP21 stabilized BRCA2 to promote DNA repair and tumor growth	Liu et al. (2017)
	MEK2	USP21-mediated stabilization of MEK2 activated MAPK/ERK signaling and thus promoted tumor growth	Li et al. (2018)
	None reported	Upregulated hsa_circ_0039053 in tumor tissues and cells positively regulated USP21 expression through sponging miR-637	Yang et al. (2020)
	None reported	One hub gene reflecting the pathological progression from cirrhosis to HCC	Lin et al. (2019)
	None reported	USP21 were significantly upregulated in HCC tissues	Yu et al. (2018)
Colorectal cancer	Fra-1	USP21 promoted colorectal cancer metastasis by stabilizing Fra-1	Yun et al. (2020)
Cholangiocarcinoma	None reported	Upregulated USP21 promotes cell proliferation and migration	Zhou et al. (2021)
Renal cell carcinoma	H2Aub	USP21 regulated IL-8 transcription and stem-cell like property	Peng et al. (2016a)
Breast cancer	p65	USP21 promoted triple negative breast cancer cell proliferation, migration and invasion	Peng et al. (2016b)
	FoxM1	USP21 promoted cell proliferation and paclitaxel resistance in basal-like breast cancer	Arceci et al. (2019)
	MARK-1/-2/-4	USP21 downregulation promoted the anchorage-independent growth of MDA-MB-231 cells	Nguyen et al. (2017)
Cervical cancer	FoxM1	USP21 regulated Hippo signaling to promote radioresistance by deubiquitylating FoxM1	Li et al. (2022)
Bladder carcinoma	EZH2	USP21 promoted cell growth and metastasis by stablizing EZH2	Chen et al. (2017b)
Diffuse large B-cell lymphoma	EZH2	Upregulation of USP21 promoted diffuse large B-cell lymphoma cell proliferation by maintaining the EZH2 level	Ma et al. (2021)
Bladder urothelial carcinoma	None reported	USP21 overexpression associated with poor outcome and related to chemoresistance in patients with metastatic urothelial carcinoma	Riester et al. (2014), Hsu et al. (2021)
Pancreatic ductal adenocarcinoma	TCF7	USP21 deubiquitylated and stabilized TCF7, thus promoting pancreas cancer cell stemness via Wnt pathway activation	Hou et al. (2019)
	MARK3	USP21 promotesd KRAS-independent tumor growth by regulation of MARK3-induced macropinocytosis	Hou et al. (2021)
Oral squamous cell carcinoma	None reported	FDG5-AS1 induced USP21 overexpression via competitively binding to miR-520b and advanced oral squamous cell carcinoma development	Liu et al. (2020)
Lung cancer	YY1	USP21/YY1/SNHG16/miR-4500 axis promoted non-small-cell lung cancer cell proliferation, migration, and invasion and *in vivo* tumor growth	Xu et al. (2020)
	PD-L1	USP21 potentially promoted immunosuppression by stabilizing PD-L1	Yang et al. (2021)
	MARK-1/-2/-4	USP21 downregulation promoted the anchorage-independent growth of A549 cells	Nguyen et al. (2017)
Gastric cancer	GATA3	USP21 promoted MAPK1 expression via stabilizing GATA3 to regulate gastric cancer cell growth and stemness	Guo Q. et al. (2021)
Osteosarcoma	None reported	Overexpression of a truncated USP21 form lacking its N-terminus inhibited U2OS cell growth	Gong et al. (2000)
Papillary thyroid carcinoma	FOXO3	RBM47-mediated stabilization of SNHG5 recruited USP21 to promote the nuclear translocation of FOXO3, resulting in activated autophagy and restrained cell proliferation	Qin et al. (2022)
Immune and Inflammation Disease			
Asthma	FOXP3 and GATA3	The imbalance of FOXP3 and GATA3 may cause decrease of Treg cells in asthma patients	Chen et al. (2018)
Schistosomiasis	None reported	USP21-deficient Tregs increased the susceptibility of mice to schistosomiasis, but reduced the degree of egg granuloma formation and liver fibrosis	Zhang et al. (2021)
Viral infection	RIG-I and STING	USP21 negatively regulated anti-viral immunity through inactivation of RIG-I and STING	Fan et al. (2014), Chen et al. (2017a)
Muscle dysfunction and associated metabolic diseases			
Obesity and type 2 diabetes	DNA-PKcs and ACLY	USP21 plays a key role in the regulation of myofibre type switch, muscle mass control, mitochondrial function, and heat generation	Kim et al. (2021)

**TABLE 4:** The sequence of Ub WT and selected Ub variants targeting USP21.

	Sequence (54–71)^a^	IC_50_ (nM)	References
WT	RTLSDYNIQKESTLHLVL	18,000	Sun et al. (2016)
Ubv21.4	RTLSDYNIQKWSTLFLLL	2.4 and 9.4	Ernst et al. (2013), Sun et al. (2016)
Ubv1	YPLAWYDITKFATLFLTG	13.9	Sun et al. (2016)
Ubv2	WTLAYYDIYRNATLFLSA	9.9	Sun et al. (2016)
Ubv4	YTLEYYNITKHATLFLVL	40.4	Sun et al. (2016)
Ubv10	ATAADYDIGQNATLFLTS	4.4	Sun et al. (2016)

a Ub WT, and selected Ub variants consists of 76 aa, we here show aa sequences from 54 to 71.

**TABLE 5:** Small molecule inhibitors of USP21.

	Structure	IC_50_ (μM)	References
Disulfiram	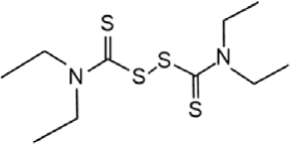	3.7 ± 0.4	Lin et al. (2021)
6-Thioguanine	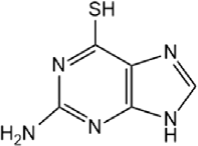	22.7 ± 0.4	Lin et al. (2021)
Spongiacidin C	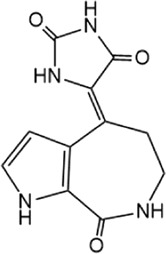	16.6 ± 2.8	Yamaguchi et al. (2013)
KYT-36	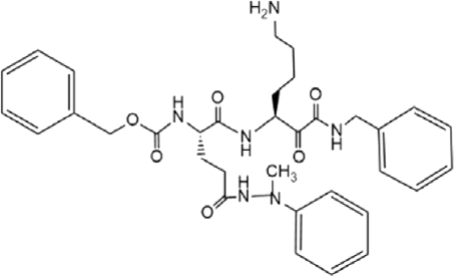	None reported	Nakagawa et al. (2008)
Cryptotanshinone	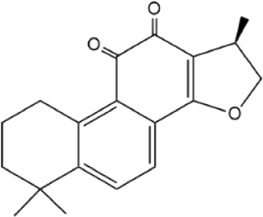	None reported	Jin et al. (2016)
SAHA	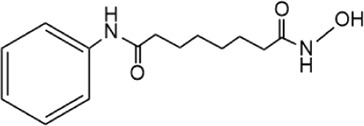	None reported	Lee et al. (2021)
MS-275	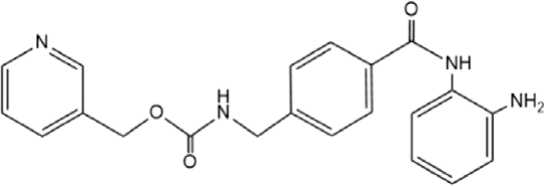	None reported	Lee et al. (2021)

## Properties OF USP21

### Chromosomal Location, Subcellular Localization, and Isoforms of USP21

The human *USP21* gene was initially mapped to chromosome 1q21 and 1q22 and later to 1q23.3 (Gong et al., 2000; Smith and Southan, 2000; Riester et al., 2014). Northern blot analysis showed that *USP21* mRNA was generally expressed at moderate levels except in the heart, pancreas, brain, skeletal muscle, and testes, where higher mRNA levels are detected, and in the lungs, small intestine, colon (mucosal lining), and peripheral blood leukocytes, where expression is very low (Gong et al., 2000; Smith and Southan, 2000). Subcellular localization of USP21 labeled with fluorescent proteins suggested that USP21 was mainly localized in the cytoplasm and showed a unique binding pattern with microtubules and centrosomes (Garcia-Santisteban et al., 2012; Urbe et al., 2012). In contrast, the discovery of USP21 substrates in the nucleus suggests that USP21 also exists in this organelle (Nakagawa et al., 2008; Zhang et al., 2013; Tao et al., 2014; Hou et al., 2019). Although appended fluorescent protein tags may interfere with the correct localization (Urbe et al., 2012), this discrepancy could be predominantly reconciled with the capacity of USP21 to shuttle between the nucleus and cytoplasm.

**FIGURE 2:**
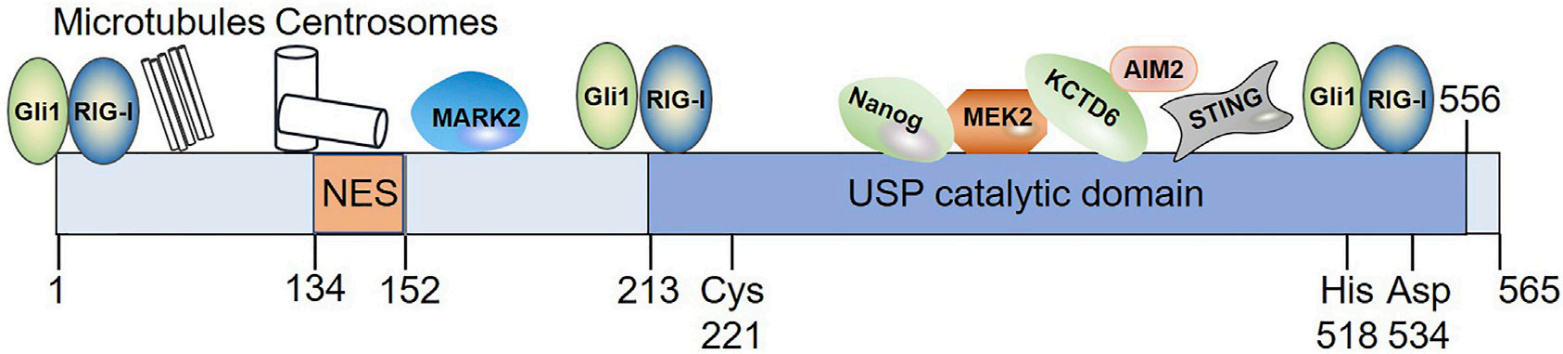
Diagram illustrating the domain organization of USP21 as well as its interaction with substrates whose binding domain in USP21 have been defined. Among these interacting partners, Gli1 and RIG-I bind to multiple regions of USP21. NES spans residues 134–152; Cys221, His518, and Asp534 indicates the position of its catalytic residues. NES, nuclear export sequence.

Combined with web-based prediction programs and experimental validation, sequence motif encompassed within amino acids 134–152 of the N-terminus of USP21 was revealed to be a physiologically relevant nuclear export sequence (NES) and mediated CRM1-dependent nuclear export of this enzyme (Figure 2). However, the nuclear localization signals (NLSs) of USP21 remain to be elucidated (Garcia-Santisteban et al., 2012). Consistent with this result, a mutation (L144H) found in a large intestine carcinoma inactivated the activity of USP21 NES, leading to its marked accumulation in the nucleus (Prieto et al., 2016). Whether this phenomenon is the main cause of this type of tumor deserves further study. Furthermore, NES and NLS are not determinants of protein subcellular localization. Protein post-translational modifications and binding to other proteins also function as switches that regulate protein subcellular localization (Dostie et al., 2000; Gabay-Maskit et al., 2020; Aggarwal et al., 2021; Navarro-Lerida et al., 2021). Taking USP4 as an example: AKT-mediated phosphorylation relocates nuclear USP4 to the cytoplasm and membrane (Zhang et al., 2012), while the binding of USP4 to Sart3 efficiently promotes its nuclear translocation (Song et al., 2010). As discussed below, USP21 is also regulated by phosphorylation and various binding partners have been identified. We speculate that similar mechanisms, such as post-translational modifications and associations with certain regulators, may be involved in the regulation of subcellular localization of USP21.

USP21 is highly conserved among species (Nakagawa et al., 2008). To date, apart from the full-length USP21 (565 aa for human USP21 and 566 aa for mouse Usp21), Okuda et al. identified a Usp21 short variant (Usp21SV) in mice, with alternative splicing in exon 2 leading to the omission of 87 amino acids in the N-terminus of Usp21 long isoform. Notably, Usp21SV lacks NES and thus localizes more in the nucleus than the Usp21 long isoform (Okuda et al., 2013).

### Structure and Activities of USP21

Compared with the majority of DUBs, the structure of USP21 is relatively simple (Komander et al., 2009). The N-terminal region (1–212 aa) of USP21 is intrinsically disordered, whereas the C-terminus contains a catalytic domain (Figure 2) (Ye et al., 2011; Urbe et al., 2012). Fewer studies are available on substrate-binding regions in USP21; however, they revealed that both the amino terminus and C-terminal catalytic domains were responsible for substrate recognition (Figure 2). The N-terminus of USP21 is required for microtubule-affinity regulating kinase 2 (MARK2), microtubule, and centrosome association (Urbe et al., 2012), while the C-terminal catalytic domain mediates its interaction with mitogen-activated protein kinase kinase 2 (MEK2), potassium channel tetramerization domain containing 6 (KCTD6), Nanog, stimulator of interferon (IFN) genes (STING), and absent in melanoma 2 (AIM2) (Heride et al., 2016; Liu et al., 2016; Chen et al., 2017a; Li et al., 2018; Hong et al., 2021). In addition, Gli1 and RIG-I bind to multiple regions of USP21 (Heride et al., 2016; Chen et al., 2017a).

Like most DUBs, USP21 can hydrolyze the isopeptide bond between Ub and target proteins, and the activity of USP21 is critically dependent on the conserved catalytic triad consisting of Cys 221, His 518, and Asp 534 (Figure 2) (Nakagawa et al., 2008). At least three Ub-binding sites, S1, S1’, and S2, have been found in USP21 (Ye et al., 2011; Mevissen and Komander, 2017). In particular, the S2 binding site seems not to be directly involved in the catalytic efficiency, but it serves to target the enzyme to polyubiquitin chains (Ye et al., 2011). For Ub, Phe 4, Thr 12, Thr 14, and Arg 72 are responsible for recognition by USP21 (Ye et al., 2011; Shin et al., 2017). *In vitro*, USP21 efficiently cleaves the ubiquitin chains of different linkages and is employed as research tool to analyze Ub-linkage type and architecture using Ub chain restriction (Ye et al., 2011; Hospenthal et al., 2015). In addition, USP21 displays promiscuous *in vivo* activity against Ub chain types (Tables 1, 2). USP21 can remove monoubiquitylation and K27-, K48-, and K63-type ubiquitin chains depending on the cellular context and the substrate. For instance, USP21 acts as a negative regulator of RIG-I and receptor-interacting protein 1 (RIP1) by cleaving K63 linkages, whereas it deubiquitylates and stabilizes GATA3 by removing K48-linked Ub chains (Xu et al., 2010; Zhang et al., 2013; Fan et al., 2014).

Furthermore, USP21 is not only specific for Ub conjugates, but also exhibits cross-reactivity toward some Ub-like modifications (Gong et al., 2000; Ye et al., 2011; Shin et al., 2017). For instance, USP21 can disassemble interferon-stimulated gene 15 (ISG15) from physiologically relevant targets due to the presence of the S2 site in USP21 and the same C-terminus of ISG15 compared with Ub. Nevertheless, kinetic studies, using Ub-AMC and ISG15-AMC as substrates revealed that the DUB activity of USP21 was approximately 70 times higher than its deISGylating activity (Ye et al., 2011). Regrettably, no cellular substrates whose ISGylation could be reversed by USP21 have been reported, and whether or not the deISGylation activity is physiologically relevant remains elusive. In addition, it is noteworthy that the hydrolysis activity of USP21 against neural precursor cell expressed developmentally downregulated 8 (NEDD8) conjugates appears to be inconsistent. Gong et al. reported that USP21 can remove higher-molecular-weight NEDD8 conjugates in COS cells co-expressing NEDD8 and truncated USP21 (residues 185–565) (Gong et al., 2000). However, truncated USP21 (residues 196–565 and residues 201–560) cannot hydrolyze the NEDD8-AMC or purified NEDD8-His, which act as useful substrates for the study of deNEDDylating activity, nor can it be modified with NEDD8-based suicide probes, which can react with enzymes with deNEDDylating activity (Ye et al., 2011; Shin et al., 2017). Interestingly, USP21 was active on the NEDD8 suicide probe and NEDD8-His, in which four residues were mutated to their Ub equivalents (NEDD8 K4F, E12T, E14T, and R72A) (Ye et al., 2011; Shin et al., 2017). These differences in the deNEDDylating activity of USP21 may arise from the different USP21 amino acid sequences used or the biochemical analyses employed with various forms of substrates.

## USP21 and Signaling Pathways

### NF-κB Signaling

**FIGURE 3:**
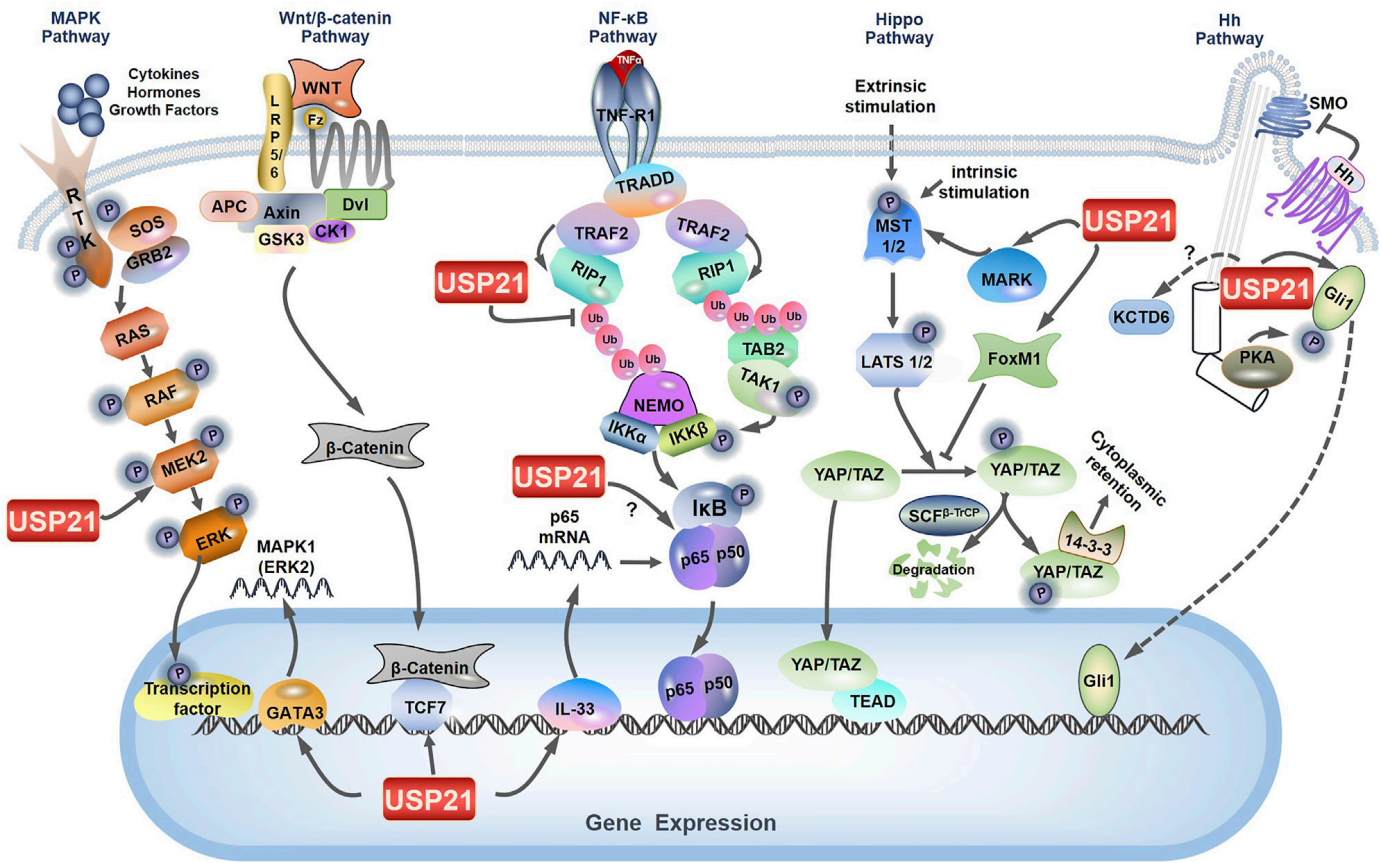
Role of USP21 in multiple signaling pathways. The figure depicts the key components and signal transduction cascade reactions in MAPK/ERK signaling, NF-κB signaling, Wnt/β-catenin signaling, Hippo signaling, and Hh signaling. The nodes regulated by USP21 are labeled.

NF-κB signaling consists primarily of nuclear p65/p50 and is activated following phosphorylation and subsequent degradation of the inhibitor of NF-κB (IκB) by IκB kinase (IKK) complexes. Notably, the mechanisms by which extracellular signals induce activation of NF-κB signaling vary dramatically upstream of this signaling (Hoesel and Schmid, 2013). Xu et al. established that USP21 specifically inhibits tumor necrosis factor α (TNFα)-, but not interleukin 1 (IL-1)- or TRAF6 overexpression-induced NF-κB activation (Xu et al., 2010). Investigation of the mechanism suggested that USP21 interacts with and deubiquitylates K63-linked polyubiquitylation of RIP1 (Xu et al., 2010), which serves as a platform to recruit both TGF-β activated kinase 1 (TAK1) and IKK complexes, thus facilitating TAK1 phosphorylation and activation of IKK (Figure 3) (Ea et al., 2006). However, USP21 depletion *in vivo* does not affect the stimulation of the TNF receptor, indicating that USP21 is redundant for the regulation of RIP1 activity (Pannu et al., 2015). Many DUBs, such as USP7, USP11, USP15, and USP48, are involved in NF-κB signaling at different levels (Morgan et al., 2020). Among which, A20, Cezanne, and USP4, have also been reported to remove the K63-linked polyubiquitination conjugate from RIP1, thereby downregulating NF-κB signaling as well (Wertz et al., 2004; Enesa et al., 2008; Hou et al., 2013). In particular, the carboxy-terminal domain of A20 also functions as a ubiquitin ligase that polyubiquitinates RIP1 with K48-linked ubiquitin chains, thus targeting RIP1 for proteasomal degradation (Wertz et al., 2004). Additionally, it was later found that Cezanne preferentially cleaves K11-linked polyubiquitin, suggesting that Cezanne may stabilize negative regulators of NF-κB signaling, as K11-linkages can act as proteasomal targeting signals (Bremm et al., 2010). These studies suggest that USP21 may coordinate with these DUBs to regulate TNFα-induced RIP1 deubiquitylation, thereby affecting the NF-κB pathway.

In contrast to the proposition that USP21 is a negative regulator of NF-κB signaling, Tao et al. indicated the possible role of USP21 in enhancing the activity of NF-κB signaling (Tao et al., 2014). Their results demonstrated that USP21 promotes the transcription of p65 via deubiquitylation and stabilization of IL-33, which functions as a transcriptional regulator of p65 and induces the expression of intercellular adhesion molecule-1 and vascular cell adhesion molecule-1 through its regulatory effect on the NF-κB pathway in endothelial cells (Choi et al., 2012; Tao et al., 2014). Intriguingly, the interaction between USP21 and p65 was confirmed by co-immunoprecipitation in MDA-MB-231 cells, although the regulatory effect of USP21 on p65 was not investigated (Figure 3) (Peng et al., 2016b). Thus, the modulatory effect of USP21 on NF-κB signaling may depend on the exact cellular context.

### Hippo Signaling

The Hippo signaling pathway, activated by intrinsic or extrinsic stimuli, plays a significant role in modulating cell fate and maintaining organ size (Gaspar and Tapon, 2014; Yu et al., 2015; Santinon et al., 2016). MST1/2 kinases phosphorylate and activate LATS1/2 kinases, which subsequently phosphorylate the transcriptional co-activators YAP and TAZ, leading to cytoplasmic sequestration or degradation by the E3 ligase SCF^β-TrCP^ (Liu et al., 2010; Zhao et al., 2010; Wang et al., 2011). Accordingly, the activation state of the Hippo signaling pathway is inversely related to the activity of the transcriptional co-activators YAP and TAZ. In addition, the LKB1-MARKs cascade was identified to function upstream of MST/LATS kinases and enhance their enzyme activity (Mohseni et al., 2014). Several DUBs can regulate Hippo signaling in a different manner. USP1, USP26, and MINDY1 have been reported to regulate the stability of YAP or TAZ, while whose nuclear localization is regulated by USP9X and OTUD1 through modulating the stability of LATS kinases or their non-proteolytic ubiquitylation (Toloczko et al., 2017; Yao et al., 2018; Luo et al., 2021; Tang et al., 2022; Yuan et al., 2022).

Overexpression of USP21 suppresses the transcriptional activity of YAP by regulating the stability of MARK-1, -2 and -4 (Nguyen et al., 2017), which indicated that MARKs are interacting partners of USP21 (Figure 3) (Sowa et al., 2009; Urbe et al., 2012). Moreover, a recent study provides other contradictory evidence that USP21 enhances the nuclear translocation and transcriptional activity of YAP by interacting with and stabilizing Forkhead box protein M1 (FoxM1), a transcription factor (Li et al., 2022). As previously described by Sun et al., FoxM1 promoted nuclear translocation and transcriptional activity of YAP by inhibiting its phosphorylation; however, the underlying mechanism remaines elusive (Figure 3) (Sun et al., 2020). Taken together, the discrepancies in USP21 modulation of the Hippo signaling pathway may be explained by differences in cell context-specific regulation.

### Wnt/β-Catenin Signaling

The canonical Wnt/β-catenin signaling pathway is transduced by Wnt receptors of the Frizzled family and stabilizes β-catenin, which then translocates to the nucleus as a transcriptional coactivator of T-cell factor/lymphoid enhancer-binding factor (TCF/LEF) transcription factors (MacDonald et al., 2009). As summarized by Park et al., deubiquitylation induced by various DUBs, such as USP2a, USP4, and USP6, is a major mechanism for regulating the stability of Wnt signaling components (Park et al., 2020). USP21 has been shown to enhance Wnt signaling through deubiquitylation and stabilization of the long isoform of transcription factor TCF7, which belongs to the TCF/LEF family and mediates oncogenic activation of Wnt signaling (Figure 3) (Shiokawa et al., 2017; Hou et al., 2019). Additionally, two other studies demonstrated that deubiquitylation and stabilization of FoxM1 by USP5 and USP28 contribute to its nuclear accumulation, which facilitates the recruitment of β-catenin to the Wnt target gene promoter and activates the Wnt signaling pathway (Chen et al., 2016; Chen L. et al., 2021). Given that FoxM1 has also been identified as a substrate of USP21 (Arceci et al., 2019; Li et al., 2022), it is reasonable to presume that the USP21-FoxM1 axis is involved in the regulation of the Wnt/β-catenin pathway.

### Mitogen-Activated Protein Kinase (MAPK)/Extracellular-Signal-Regulated Kinase (ERK) Signaling

The MAPK signaling cascades represent a set of membrane-to-nucleus signaling pathways that lead to the phosphorylation and activation of transcription factors and are organized into a three-tiered hierarchy. In this hierarchy, MAPK kinase kinase (MAPKKK) is activated to phosphorylate and activate MAPK kinase (MAPKK), which in turn phosphorylates and activates MAPK. Three major MAPK cascades have been identified in mammals: MAPK/ERK, MAPK/c-Jun N-terminal or stress-activated protein kinases (JNK), and MAPK/p38 (Pearson et al., 2001; Asomugha et al., 2010). Recently, DUBs and E3 ligases targeting the MAPK signaling pathway are well discussed (Park and Baek, 2022). In hepatocellular carcinoma cells, USP21 directly interacts with and stabilizes MEK2 by decreasing its K48-linked polyubiquitination, thus activating the MAPK/ERK branch, where MAPKKK is Raf, MAPKK is MEK, and MAPK is ERK (Figure 3) (Rushworth et al., 2006; Li et al., 2018). In addition, Guo et al. demonstrated that USP21 also promotes the expression of MAPK1 (also known as ERK2) by binding to transcription factor GATA3 (Figure 3) (Guo Q. et al., 2021). These studies indicate that USP21 functions as a positive regulator for the MAPK/ERK signaling.

### Hedgehog (Hh) Signaling

Hh signal transduction is modulated by DUBs at multiple steps along the pathway (Zhang and Jiang, 2021). Current studies indicate that the regulatory effects of USP21 on Hh signaling are intricate. USP21 is required for effective microtubule regrowth from centrosomes, and its depletion restrains the generation of primary cilia (Urbe et al., 2012), a specialized microtubule-based organelle templated by the centrosome and involved in the initiation of Hh signaling in vertebrates (Heride et al., 2016; Stoufflet et al., 2020). Briefly, the binding of Hh ligands to Patched (PTCH) triggers their endocytosis and results in de-repression of Smoothened (SMO), thus favoring the activation of downstream transcription factors Gli1, Gli2, and Gli3, which consequently modulate the expression of Hh target genes involved in key cellular processes such as cell cycle, migration, and metabolism (Ryan and Chiang, 2012). Another mechanism by which USP21 modulates Hh signaling focuses on its regulation of Gli1. On the one hand, USP21 can regulate the stability of Gli1 like USP7 and USP48 (Zhou et al., 2015; Heride et al., 2016; Zhou et al., 2017). USP21 binds to and stabilizes Gli1, and knockdown of USP21 decreases the transcriptional activity of Gli1 (Figure 3). On the other hand, USP21 overexpression similarly represses Gli1 transcriptional activity, which is at odds with increased Gli1 protein levels. Further investigations indicated that this is a consequence of USP21 overexpression simultaneously promotes Gli1 localization and phosphorylation by protein kinase A (PKA) at the centrosome (Figure 3) (Heride et al., 2016). As previously deciphered, phosphorylation of Gli1 at Thr 374 by PKA retains Gli1 in the cytoplasm, thereby regulating its transcriptional activity (Sheng et al., 2006).

USP21 is also associated with KCTD6, a negative regulator of Hh signaling, by targeting HDAC1 for proteasomal degradation (Figure 3) (De Smaele et al., 2011; Heride et al., 2016). Although USP21 does not affect KCTD6 protein levels, co-expression of KCTD6 with USP21 counteracts its capacity to stabilize Gli1 because KCTD6 and Gli1 competitively bind to the catalytic domain of USP21 (Heride et al., 2016). Collectively, these studies suggest that appropriate protein levels of USP21 may be crucial for maintaining optimal Hh signaling.

## Role OF USP21 IN Diverse Biological Processes

### Epigenetic Regulation

Diverse post-translational modifications of histones such as ubiquitylation, methylation, acetylation, and phosphorylation, are well-established molecular carriers of epigenetic information (Barbour et al., 2020). Furthermore, interplay and crosstalk between distinct histone modifications form a complicated web of gene regulation, termed the histone code (Wong et al., 2015). Histone H2A monoubiquitylation at lysine 119 (H2Aub) is one such modification generally associated with gene repression; its hydrolysis by several DUBs, including USP16, USP21, and BAP1, is an important pattern to regulate myriad gene transcription (Cao and Yan, 2012; Barbour et al., 2020). Based on microarray expression data from the regenerating liver, Nakagawa et al. first identified that USP21 was upregulated after partial hepatectomy and then catalyzed the hydrolysis of nucleosomal H2Aub, which facilitates di- and trimethylation of H3K4 and the transcriptional initiation of various genes closely associated with liver regeneration (Nakagawa et al., 2008). USP21 binds to the promoter of IL-8 and promotes its transcription by decreasing H2Aub with concomitant H3K4me3 elevation (Peng et al., 2016a). Similarly, USP21 was recruited to gene promoters by Nanog to deubiquitinate H2Aub, followed by increased enrichment of H3K4me3, thus promoting Nanog-mediated gene expression in mouse embryonic stem cells (Jin et al., 2016). In a nitrosodiethylamine (NDEA)-induced liver cancer model and hepatocellular carcinoma CL38 cells, USP21 markedly increased and downregulated H2Aub. Along with decrease in H2Aub levels, a decrease in H4ac and an increase in mitotic mark H3S10p levels were observed (Bhattacharya et al., 2016). USP21 also improves the reprogramming of gene expression through its deubiquitylation activity against H2Aub, thus reducing resistance to transcriptional reprogramming in mouse nuclear transfer embryos (Jullien et al., 2017). Consistent with these results, a recent study implied that USP21 is likely crucial for somatic cell nuclear transfer reprogramming (Deng et al., 2021). Furthermore, both recombinant mouse Usp21 variants activated transcription by deubiquitylating H2Aub *in vitro* (Okuda et al., 2013). In addition, USP21 mitigated the stabilization effect of H2Aub on the nucleosome, which may favor the passage of RNA or DNA polymerases through the nucleosome barrier during gene transcription or replication (Xiao et al., 2020).

Although two studies found that USP21 maintained the protein level of EZH2, the enzymatic catalytic subunit of the polycomb repressive complex 2 that can modulate gene expression by trimethylating K27 on histone 3 (H3K27), in which the impacts of USP21 on H3K27me3 level were not tested (Chen et al., 2017b; Ma et al., 2021). Reciprocally, USP21 expression dramatically elevated H3K9 methylation without affecting H3K4 methylation or H3K27 acetylation on the promoter of cyclin T1, which resulted in decreased cyclin T1 expression (Gao et al., 2021). Whether this correlates with the hydrolysis of H2Aub remaines unclear. Thus, USP21 plays a dual role in controlling gene expression through epigenetic regulation. However, little is known about the mechanism underlying how USP21 regulates epigenetic modulation that controls specific gene expression associated with chromatin contexts.

### Centrosome and Microtubule-Associated Functions

USP21 has been characterized as a unique DUB that is directly associated with both microtubules and centrosomes. It therefore plays a significant role in the governance of microtubule- and centrosome-related physiological processes, including the regeneration of the microtubule network after cold-induced depolymerization, the formation of primary cilia, and neurite outgrowth induced by nerve growth factor (Urbe et al., 2012). Although several microtubule-associated proteins, such as MARKs and CKAP5, were also identified as interacting partners of USP21, USP21 knockdown did not affect the expression and ubiquitylation levels of these proteins (Urbe et al., 2012). However, later studies demonstrated that USP21 could regulate YAP transcriptional activity and cytoskeleton-based cellular process macropinocytosis by regulating the stability and ubiquitylation of MARKs (Nguyen et al., 2017; Hou et al., 2021). To our knowledge, sever other DUBs, including CYLD, UCHL1, and BRCC36, have also been identified to be associated with microtubules, establishing the links between deubiquitylation modification and microtubule cell biology (Stegmeier et al., 2007; Gao et al., 2008; Bheda et al., 2010; Yan et al., 2015).

### DNA Repair

Liu et al. suggested that USP21 participates in homologous recombination (HR) repair upon DNA double-strand breaks (DSBs). By deubiquitinating and stabilizing BRCA2, USP21 promotes efficient RAD51 accumulation at DSBs. The formation of RAD51-single-stranded DNA filaments initiates homology search and strand invasion to complete HR. Consequently, USP21 depletion reduced the efficiency of HR, leading to increased DNA damage load and concomitant impairment of hepatocellular carcinoma (HCC) cell survival (Liu et al., 2017). Current studies demonstrate that other DUBs are also involved in BRCA2-RAD51 axis, as well as the rest of core factors in DNA repair pathways (Li and Yuan, 2021). For example, UCHL3 is phosphorylated and activated by ATM following DNA damage, which in turn deubiquitinates RAD51 and promotes the interaction between RAD51 and BRCA2 (Luo et al., 2016). Together, these studies indicate the important role of DUBs in repairing DNA DSBs (Li and Yuan, 2021).

### Antiviral Response and Immune Regulation

USP21 functions as a negative regulator of the innate immune response to RNA or DNA virus infection by suppressing the expression and production of type I IFNs by cleaving K63-linked polyubiquitin chains from RIG-I or K27/63-linked polyubiquitin chains from STING (Fan et al., 2014; Chen et al., 2017a; Wu et al., 2021). In addition to USP21, multiple DUBs targeting RIG-I, STING, and other components of type I IFN signaling have been recently reviewed. For instance, at least 9 DUBs, including USP21, have been identified to counteract the K63-linked polyubiquitylation of RIG-I (Qian et al., 2021). Moreover, p38 activated by DNA virus induces phosphorylation of USP21 at Ser538 (Ser539 in mouse Usp21) and further enhances the binding of USP21 to STING, but not RIG-I, indicating that phosphorylation of USP21 by p38 differentially regulates host defense against DNA and RNA virus infection (Chen et al., 2017a). Previous studies have suggested that USP21 depletion enhances the immune defense against a set of viruses, thus reducing their replication *in vitro* and *in vivo* (Fan et al., 2014; Chen et al., 2017a; Wu et al., 2021). The recent identification of USP21 as the DUB responsible for the stabilization of AIM2 and immediate AIM2 inflammasome activation upon DNA stimulation further complicates the role of USP21 in regulating DNA-mediated innate immunity (Hong et al., 2021). Moreover, another study indicated that USP21 inhibits human immunodeficiency virus 1 (HIV-1) replication *in vitro* by downregulating the expression of transactivator of transcription (Tat), which is essential for transcriptional elongation in HIV-1 (Gao et al., 2021). Collectively, these studies suggested a dual role for USP21 in anti-viral infections.

USP21 also plays a significant role in immune tolerance by maintaining the physiological function of regulatory T (Treg) cells, which negatively regulate immune and inflammatory responses. For instance, mice lacking *Usp21*, specifically in Treg cells, exhibited immune disorders characterized by spontaneous T-cell activation and excessive T-helper type 1 (Th1) skewing of Treg cells into Th1-like Treg cells (Li et al., 2016). However, there is a discrepancy between human and mice in the precise mechanism by which USP21 controls the stability of the Treg lineage (Zhang et al., 2013; Li et al., 2016). In humans, USP21 forms a positive feedback loop with FOXP3 and GATA3, which are two transcription factors required for maintaining the function of Treg cells in both humans and mice (Zhang et al., 2013; Li et al., 2016). GATA3 was not regulated by Usp21 in murine Treg cells (Li et al., 2016), which was in accordance with a previous study showing that Usp21 is redundant for the regulation of GATA3 during murine lymphocyte differentiation (Pannu et al., 2015). Programmed death ligand-1 (PD-L1) is a crucial immune checkpoint molecule and downregulates T-cell immune responses by binding to programmed death protein-1 (PD-1). Emerging evidence indicates that the protein stabilization of PD-L1 is regulated by several DUBs, such as USP5, USP22, and USP9X (Hu et al., 2021; Pan et al., 2021). Notably, a recent study found that USP21 could also deubiquitylate and stabilize PD-L1, suggesting a potential role of USP21 in immunosuppression (Yang et al., 2021).

### Embryonic Stem Cell Maintenance and X Chromosome Inactivation

Various DUBs play a critical role in embryonic stem cell maintenance and differentiation (Suresh et al., 2016). Employing different screening methods, three groups individually identified USP21 as a specific DUB that deubiquitylates and stabilizes Nanog, a key pluripotency factor (Jin et al., 2016; Liu et al., 2016; Kwon et al., 2017; Pei, 2017). Moreover, two of these studies further demonstrated that *Usp21* is required for maintaining the self-renewal of mouse embryonic stem cells (mESCs) and decreases upon differentiation cues; *Usp21* depletion in mESCs *in vitro* resulted in degradation of Nanog and differentiation of mESCs (Jin et al., 2016; Liu et al., 2016). However, two additional studies indicated that *Usp21* knockout mice were viable and fertile and did not show abnormalities in hematopoietic stem cell maintenance or lymphocyte differentiation, suggesting that additional DUBs may participate in the regulation of Nanog stability during early embryonic development (Fan et al., 2014; Pannu et al., 2015). A later study indicated that Bach1 facilitated the deubiquitylation and stabilization of Nanog, as well as two other major pluripotent factors, Sox2 and Oct4, through the recruitment of USP7 (Wei et al., 2019). Moreover, emerging evidence suggests that Nanog has oncogenic features such as cancer stem cell maintenance (Fatma et al., 2021; Vasefifar et al., 2022), indicating a possible role of USP21/Nanog axis in carcinogenesis.

In addition, Nanog can regulate X chromosome inactivation (XCI), a very important process in the development of normal female mammals (Navarro et al., 2008). Higher expression of USP21 and Nanog was detected in androgenetic complete hydatidiform moles (CHMs) with a 46, XX karyotype than in normal villi, suggesting that the USP21-Nanog pathway may participate in the disruption of XCI in androgenetic CHM (Chen X. et al., 2021).

### Other Biological Functions of USP21

Khan et al. provided insights into the cooperative role of USP21 in BANP, E5R, and Nac1 domain 3 (BEND3)-mediated rDNA silencing by stabilizing Tip5, a component of the nucleolar-remodeling complex (NoRC) essential for the repression of rRNA gene transcription (Khan et al., 2015). In addition to OTUD3, USP21 antagonizes the ubiquitylation of 40S ribosomal proteins and, in turn, limits the activation of the ribosome-associated quality control pathway, which recognizes stalled nascent polypeptides and targets them for degradation (Sitron and Brandman, 2019; Garshott et al., 2020). It would thus be interesting to investigate whether the expression of these two DUBs correlates with the production of aberrant proteins. Moreover, preliminary studies have suggested that USP21 might be involved in craniofacial development by regulating monoubiquitylation of the transcription factor Goosecoid (Liu et al., 2019). Despite the identification of USP21 as a potential cytoplasmic signaling effector of Down syndrome cell adhesion molecule (DSCAM) and DSCAM-Like-1 (DSCAML1), which serve significant neurodevelopmental functions, the physiological role of USP21 in signal transduction mediated by DSCAM/DSCAML1 remains to be elucidated (Sachse et al., 2019). More recently, Liu et al. showed that USP21 can remove necroptosis-induced ubiquitylation of MLKL, the key executioner in necroptosis. Wide-type USP21 fused to MLKL prevented ubiquitylation of MLKL, and this fusion could induce cell death in the absence of a necroptotic stimulus, suggesting that constitutive removal of ubiquitin from MLKL may license MLKL autoactivation (Liu et al., 2021).

## Dysregulation OF USP21 IN Diseases

### Role of USP21 in Cancer

As summarized in Table 3, many studies have validated that USP21 plays an essential role in the occurrence and progression of various cancers including HCC, colorectal cancer, and urothelial cancer. Concurrently, it is notable that conflicting functions have been reported where USP21 can act as an oncoprotein or a tumor repressor of cancer.

USP21 is highly expressed in HCC cells and correlates with poor survival in HCCs (Liu et al., 2017; Li et al., 2018; Yang et al., 2020). Several molecular mechanisms involving USP21 have been reported to be closely associated with HCC occurrence and progression. As mentioned previously, elevated Usp21 levels in HCC CL38 cells and the NDEA-induced HCC model decreased the level of H2Aub, which accounted for the increase in the mitotic marker H3S10p and the expression of lipocalin 2 with an oncogenic effect (Bhattacharya et al., 2016). Examination of whether NEDA can induce HCC in *Usp21*-deficient mice may further deepen our understanding of the oncogenic role of Usp21 at the tumor initiation stage. Liu et al. showed that USP21 interacts with and stabilizes BRCA2, a pivotal mediator of DNA repair by homologous recombination, thereby promoting DNA repair in HCC, as well as cell survival *in vitro* and *in vivo*. Notably, BRCA2 overexpression failed to fully restore HCC tumor cell growth following USP21 depletion, suggesting that USP21 may have other targets in HCC (Liu et al., 2017). A later study found that UPS21 overexpression activated ERK signaling through deubiquitylation and stabilization of MEK2, whose expression partially rescued decreased p-ERK1/2 levels, impaired cell proliferation and anchorage-independent growth, and cell cycle arrest due to USP21 knockdown in HCC cell lines (Li et al., 2018). Hsa_circ_0039053, a circular RNA, is increased in HCC tissues and cell lines, and it promotes HCC cell proliferation and invasion by positively regulating USP21 expression by sponging miR-637 (Yang et al., 2020). In line with the significant role of USP21 in HCC, the variation score of a 20 gene-based gene set including USP21 may reflect the pathological progression from cirrhosis to HCC and serve as an independent prognostic factor for recurrence-free and overall survival (Lin et al., 2019). Therefore, elucidating the synergistic effect between USP21 and other genes will help us further understand the pathogenesis of HCC. Additionally, the role of USP21 in HCC did not appear to depend on its deneddylating activity as discussed by the authors themselves (Yu et al., 2018). Taken together, these studies suggest that USP21 plays an important role in the development of HCC.

Moreover, USP21 promotes the migration and invasion of colorectal cancer by acting as a DUB for Fos-related-antigen-1 (Fra-1), a transcription factor essential for cancer progression and metastasis (Yun et al., 2020). USP21 is also critically linked to cholangiocarcinoma tumorigenesis, although the underlying mechanisms remain unclear thus far (Zhou et al., 2021). This pro-cancer effect is also seen in USP21-induced expression of IL-8 via epigenetic modulation, which promotes tumorigenic properties in renal cell carcinomas (RCC), including cell proliferation, invasion, and cancer stem cells percentage (Peng et al., 2016a). Peng et al. found that the expression of USP21 in triple-negative breast cancer (TNBC) cell lines was higher than that in other subtypes of breast cancer, which further confirmed that USP21 facilitated TNBC cell proliferation, migration, and invasion (Peng et al., 2016b). Although co-immunoprecipitation demonstrated that USP21 was associated with the NF-κB factor p65 (also known as RelA) (Peng et al., 2016b), the role of this interaction in tumorigenesis was not determined and deserves in-depth study. Subsequently, Arceci et al. revealed that USP21 amplification was related to proliferation and paclitaxel resistance in basal-like breast cancer (BLBC) by deubiquitylating and stabilizing the cell cycle transcription factor FoxM1 (Arceci et al., 2019). Notably, BLBC has a significant overlap with the TNBC subtype at the molecular level (Foulkes et al., 2010). Likewise, FoxM1 deubiquitylation by USP21 contributes to both cell growth and radioresistance in cervical cancer (Li et al., 2022). As the major cellular H3K27 trimethyltransferase, EZH2 amplification has been found in a wide range of human cancers (Kim and Roberts, 2016). By maintaining EZH2 protein levels, USP21 promotes cell proliferation and metastasis in bladder carcinoma and cell proliferation in diffuse large B-cell lymphoma (Chen et al., 2017b; Ma et al., 2021). Furthermore, high USP21 expression was associated with aggressiveness and resistance to chemotherapy in patients with bladder urothelial carcinoma (Riester et al., 2014; Hsu et al., 2021). These studies imply that USP21 not only accelerates tumor progression but also mediates resistance to some chemotherapeutic drugs or radiotherapy.

Recently, USP21 has been authenticated as a frequently amplified gene in pancreatic ductal adenocarcinoma (PDAC) with the capacity to enhance PDAC cell stemness by stabilizing TCF7 (Hou et al., 2019). Using the inducible *Kras* ^G12D^/*Trp53* ^−/−^ PDAC mouse model, the same group subsequently identified that USP21 supported the growth of oncogenic KRAS-independent PDAC by elevating MARK3-mediated macropinocytosis (Hou et al., 2021). Together, these two studies prompted the evaluation of USP21 as a novel therapeutic target, as well as a potential genetic factor that may affect responsiveness to emerging KRAS inhibitors in patients with PDAC (Crawford, 2021). Liu et al. showed that long non-coding RNA (lncRNA) FDG5-AS1 could be regarded as a competing endogenous RNA to upregulate USP21 expression combined with miR-520b, which advanced the development of oral squamous cell carcinoma (Liu et al., 2020). Furthermore, the USP21/YY1/SNHG16/miR-4500 axis has been found to assist in the development of non-small-cell lung cancer (NSCLC). USP21 deubiquitylates and stabilizes YY1, and YY1 then transcriptionally activates lncRNA SNHG16, which in turn elevates USP21 expression by targeting and suppressing miR-4500 (Xu et al., 2020). Additionally, Yang et al. uncovered the potential role of USP21 in immune escape due to the mechanism of USP21-mediated PD-L1 stabilization, and suggested the positive correlation between USP21 and PD-L1 in lung cancer, especially in lung squamous cell carcinoma, a main subtype of NSCLC (Yang et al., 2021). A previous study also suggested that USP21 depletion results in the activation of STING and protein instability of FOXP3, leading to enhanced antiviral immunity and impaired suppressive activity of Treg cells (Li et al., 2016; Chen et al., 2017a). Considering the capacity of STING to sense cytosolic tumor-derived DNA in tumor-associated immune cells, as well as the ability of Treg cells to limit anticancer immunity and promote angiogenesis (Facciabene et al., 2012; Woo et al., 2015), these results together reinforce that targeting USP21 may offer promise for antitumor immunotherapies. As mentioned above, GATA3 is a substrate of USP21 involved in immune tolerance, and this interaction was also found in gastric cancer, which further facilitated the malignant progression of gastric cancer by promoting MAPK1 (also known as ERK2) expression (Guo Q. et al., 2021). Collectively, these studies indicate that USP21 plays a critical role in the initiation and progression of various cancers, involving a set of key factors and regulatory networks, indicating that USP21 holds great promise as a potential target for the development of antitumor drugs.

However, we should remain vigilant with this conclusion, especially since the anticancer effects of USP21 have rarely been reported. The first study on the cloning of *USP21* reported that a truncated USP21 lacking its N-terminus displayed a profound inhibitory effect on U2OS cell growth (Gong et al., 2000). A later study discovered that USP21 generates antitumor activity by mediating MARKs protein turnover, resulting in the suppression of YAP/TAZ (Nguyen et al., 2017). Notably, the results of this study showed that USP21 knockdown promoted anchorage-independent growth of cancer cells, including A549 and MDA-MB-231, which seemed to be inconsistent with several studies indicating the positive role of USP21 in cell proliferation, migration, and invasion of A549 and MDA-MB-231 (Urbe et al., 2012; Peng et al., 2016b; Arceci et al., 2019; Xu et al., 2020). A recent study showed that RBM47 inhibited proliferation in papillary thyroid carcinoma by stabilizing the lncRNA SNHG5, which in turn acted as a scaffold, binding with USP21 to regulate the expression of FOXO3, implying that USP21 is required for the tumor-suppressive role of RBM47 (Qin et al., 2022). Therefore, the tumor-suppressive effects of USP21 need to be confirmed in further studies.

### Immune-Related Diseases

Increased expression of USP21 has been found in Treg cells of asthma patients (Zhang et al., 2013; Chen et al., 2018); the imbalance of FOXP3 and GATA3 is an important cause of pathogenic alteration of Treg cells in asthma patients (Chen et al., 2018). Given that USP21 has been confirmed to regulate the stability of both FOXP3 and GATA3 (Zhang et al., 2013; Li et al., 2016), the mechanism by which USP21 imbalances GATA3 and FOXP3 remains obscure (Chen et al., 2018). This may be clarified by investigating whether GATA3 and FOXP3 compete in the same domain of USP21 and the affinity of USP21 toward these two substrates. An additional study uncovered the multifaceted effects of USP21 in Treg cell-mediated regulation of immune interactions between *Schistosoma* and its host, suggesting the potential role of USP21 in regulating liver fibrosis in patients with schistosomiasis (Zhang et al., 2021). In addition, USP21 depletion contributed to the significant production of IFNs by activating the RIG-I or STING pathway, raising the possibility that USP21 inhibitors may enhance immune responses against virus infection (Fan et al., 2014; Chen et al., 2017a).

### Muscle Dysfunction and Associated Metabolic Diseases

USP21 was initially found to be highly expressed in several tissues including skeletal muscle (Gong et al., 2000; Smith and Southan, 2000). Kim et al. recently identified the regulatory role of USP21 in balancing fuel storage and energy expenditure in skeletal muscle. Their results demonstrated that DNA-dependent protein kinase catalytic subunit (DNA-PKcs) and ATP citrate lyase (ACLY) are specific substrates of USP21 that regulate the activity of AMPK, an important energy sensor. Notably, USP21 expression is upregulated in the skeletal muscle of a diabetic patient and high-fat diet-induced obese mice. Collectively, this study provides evidence that USP21 and its downstream substrate network are potential targets for the treatment of muscle dysfunction and associated metabolic diseases, such as obesity and type 2 diabetes (Kim et al., 2021).

## Cellular Regulation OF USP21

**FIGURE 4:**
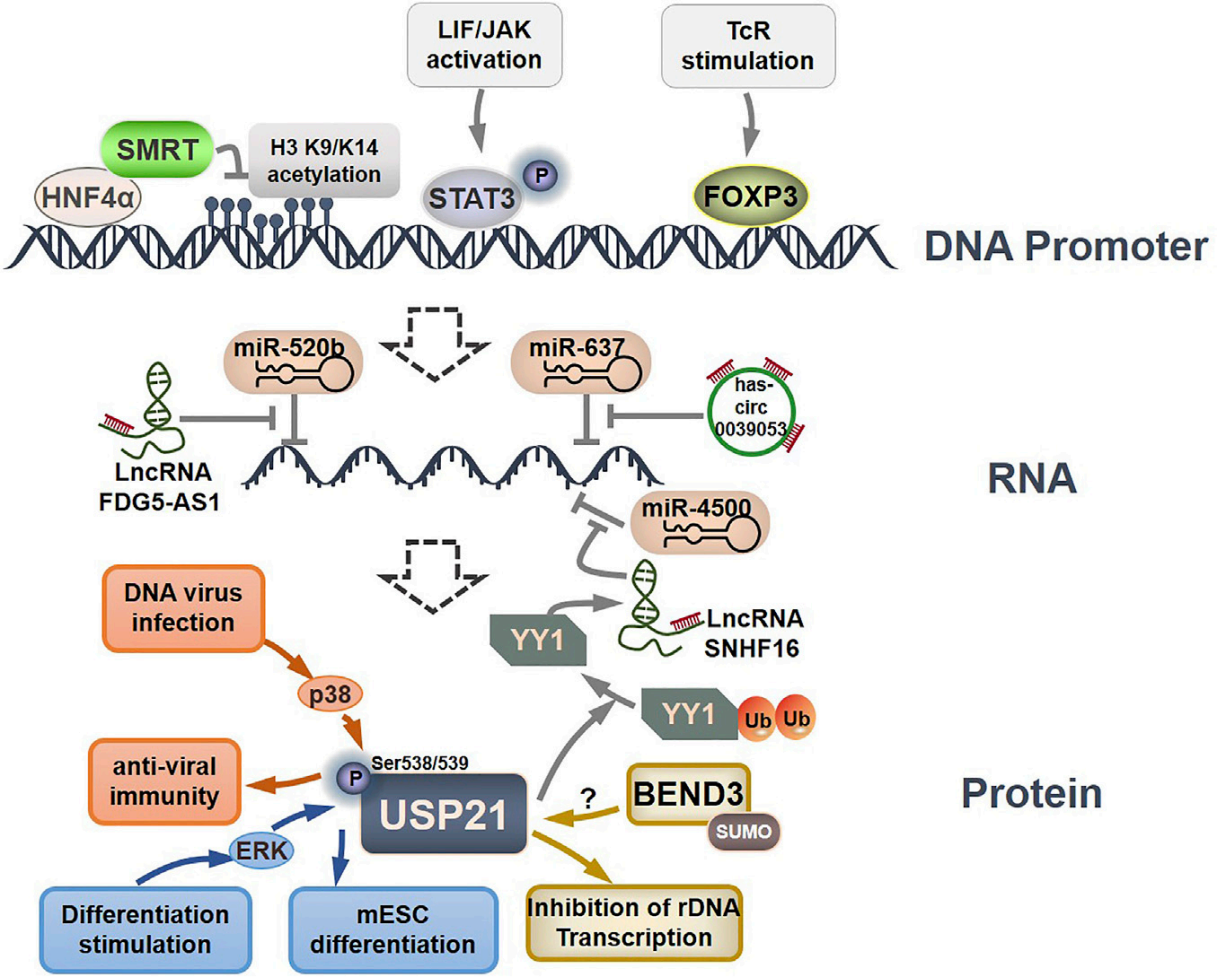
Regulation of USP21 expression. HNF4α, STAT3, and FOXP3 can directly bind to the USP21 promoter to modulate the transcription of USP21. Several lncRNAs regulate USP21 expression by sponging miRNAs. ERK and p38 phosphorylated USP21 to regulate its substrate binding affinity, and SUMOylated BEND3 stabilize USP21 in an indistinct manner.

Currently, our understanding of the regulatory mechanisms of USP21 expression, stability, and function is limited (Figure 4). At the transcriptional level, HNF4α was first reported to suppress *USP21* transcription by directly recruiting SMRT to its promoter region, leading to reduced acetylation of histone H3 K9 and K14 (Ungaro et al., 2010). A potential FOXP3 binding site within the -352 and -346 regions, as well as two potential STAT3-binding sites within the −836 to −828 and −3,054 to −3,033 regions, were subsequently identified in the *USP21* promoter (Zhang et al., 2013; Jin et al., 2016). FOXP3 can specifically bind to the *USP21* promoter and activate its transcription after T-cell receptor (TcR) stimulation (Zhang et al., 2013). Notably, phosphorylation of STAT3 at Tyr 705, activated by LIF/JAK signaling, is required for the positive regulation of USP21 in mESCs (Huang et al., 2014; Jin et al., 2016). Moreover, circular RNA hsa_circ_0039053 and lncRNAs, including FDG5-AS1 and SNHG16, can upregulate USP21 by downregulating specific miRNAs (Liu et al., 2020; Xu et al., 2020; Yang et al., 2020). Specifically, USP21 was negatively regulated by miR-637 in HCC, and this inhibition could be reversed by hsa_circ_0039053, which acts as a sponge to prevent miR-637 from associating with USP21 mRNA (Yang et al., 2020). In contrast, FDG5-AS1 and SNHG16 function as miRNA sponges for miR-520b and miR-4500, leading to increased expression of USP21 in oral squamous cell carcinoma and NSCLC, respectively, (Liu et al., 2020; Xu et al., 2020).

To date, the sole post-translational modification linked with USP21 regulation is phosphorylation, which in turn affects its affinity for substrate proteins. Phosphorylation of mouse Usp21 at Ser539 by ERK1 in mESCs in response to differentiation stimuli blocked the interaction between Usp21 and Nanog, which consequently resulted in Nanog degradation and mESC differentiation (Jin et al., 2016). In contrast, p38-mediated phosphorylation of USP21 at Ser538 (Ser539 in mouse Usp21) in response to DNA virus infection promotes the binding of USP21 to STING, thus leading to the inactivation of STING and decreased efficiency of antiviral immunity (Chen et al., 2017a).

On a final note, Khan et al. found that SUMOylated BEND3 could stabilize USP21. However, the underlying mechanism is not fully understood and warrants further study (Khan et al., 2015). These findings indicate that the expression, substrate-binding affinity, and stability of USP21 are tightly controlled by different mechanisms under different physiological and pathological conditions. Thus, more studies are urgently needed to establish a clear regulatory network for USP21.

## Inhibitors OF USP21

Studies carried out in the past few decades have revealed the critical roles of DUBs in various diseases, especially cancer, providing sufficient evidence for the development of these molecules as potential pharmaceutical targets, and considerable progress has been made in the development of inhibitors against several DUBs such as USP1 and USP7 (Harrigan et al., 2018; Chen S. et al., 2021; Nininahazwe et al., 2021). Nevertheless, progress in the development of USP21 inhibitors remains limited. By integrating multiple strategies and technologies, several studies have isolated multiple Ub variants, such as Ubv.21.4 and Ubv10, which selectively bind and potently inhibit USP21 with low nanomolar IC_50_ in in vitro proteolysis assays (Table 4) (Ernst et al., 2013; Leung et al., 2016; Sun et al., 2016; Sun and Kim, 2017). Moreover, overexpression of Ubv.21.4CΔ2, the truncated Ubv.21.4 form lacking the last two glycines, also efficiently inhibited cellular USP21 activity, blocking the deubiquitylation of RIP1 and STING by USP21 (Ernst et al., 2013; Chen et al., 2017a). Furthermore, Ub variant inhibitors targeting other DUBs, such as USP8, USP2a, OTUB1, STAMBP, STAMBPL1, and USP28, have been identified, implying that Ub variants are suitable to serve as conducive genetic probes for investigating and modulating ubiquitin system function (Ernst et al., 2013; Guo Y. et al., 2021; Veggiani et al., 2022).

Disulfiram, an anti-alcohol abuse drug actively being repurposed for cancer, and 6-Thioguanine (6 TG), a clinical drug for acute myeloid leukemia, were recently identified as competitive inhibitors of USP21 (Table 5). Co-treatment with disulfiram and 6 TG exhibited a synergistic effect of USP21 inhibition, indicating that these two drugs may modify cysteine residues in different regions of USP21 (Lin et al., 2021). This encourages the evaluation of combination treatment with disulfiram and 6 TG for USP21 dysregulated diseases including cancer. Furthermore, disulfiram and 6 TG synergistically inhibited the enzymatic activity of USP2 (Lin et al., 2021), but whether these two compounds suppress the activity of other DUBs remains to be studied further. Moreover, two other non-selective small-molecule inhibitors of USP21, spongiacidin C and KYT-36, have been reported (Table 5). However, they do not have the expected biological functions, such as cytotoxicity and transcriptional inhibition (Kadowaki et al., 2004; Nakagawa et al., 2008; Yamaguchi et al., 2013).

In addition, the STAT3 inhibitor cryptotanshinone and HDAC inhibitors (SAHA and MS-275) have been reported to downregulate the expression of USP21 (Table 5) (Jin et al., 2016; Lee et al., 2021). Although the antitumor activity of cryptotanshinone and HDAC inhibitors has been studied extensively, the role of USP21 in antitumor activity remains unclear (Ashrafizadeh et al., 2021; Ruzic et al., 2022). In summary, highly potent and specific inhibitors targeting USP21 urgently need to be discovered and developed, which may serve as promising agents for the treatment of multiple forms of cancer and other diseases.

## Conclusion and Future Perspectives

Numerous efforts over the last decade have greatly enriched our understanding of USP21. The identification of a wide range of USP21 substrates revealed the momentous and multifaceted role of USP21 in physiological and pathological states, especially in tumorigenesis, highlighting that USP21 is emerging as an appealing target for the therapy of many correlative diseases. Nonetheless, the finding that USP21 appears to play a dual role in several biological processes and pathological conditions emphasizes the need to evaluate the role of USP21 in different environments and diseases. For example, a majority of studies indicated that USP21 supported proliferation and progression, as well as potential immunosuppression in distinct cancer types, whereas the suppressive roles of USP21 have also been reported. Thus, future studies should focus on deciphering which cancers and other diseases are likely to benefit from USP21 inhibition. Meanwhile, we should be alert to the adverse effects of immune system activation that may be caused by USP21 inhibition. This is because USP21 maintains the suppressive activity of Treg cells and decreases the production of IFNs (Fan et al., 2014; Li et al., 2016; Chen et al., 2017a). Several other critical issues remain to be resolved. For instance, key factors regulate the shuttling of USP21 from the cytoplasm to the nucleus. It is not known how USP21 specifically regulates its diverse downstream signaling substrates in different contexts. In addition, the upstream regulators of USP21 are not completely understood.

Therefore, the regulation and function of USP21 in normal and pathological states still require further investigation, which will provide deep insights into its activity and specificity, strengthening the foundation for the development of selective USP21 inhibitors as a viable strategy to treat cancer and other diseases. Moreover, there is only a limited exploration of the inhibition of USP21, mainly represented by the small numbers, low activity and selectivity of inhibitors, and undefined or negligible pharmacological effects. Thus, more effective inhibitors targeting USP21 are required. This will advance the understanding of USP21 function and manipulation of USP21 for therapeutic benefit, paving the way for the development of USP21 as a potential therapeutic target.

## Glossary

AA: amino acid
AIM2: absent in melanoma 2
ACLY: ATP citrate lyase
BEND3: BANP, E5R, and Nac1 (BEN) domain 3
BLBC: Basal-like breast cancer
CHMs: complete hydatidiform moles
DUBs: Deubiquitylating enzymes
DSBs: DNA double-strand breaks
DNA-PKcs: DNA-dependent protein kinase catalytic subunit
DSCAM: Down syndrome cell adhesion molecule
DSCAML1: DSCAM-Like-1
ERK: extracellular-signal-regulated kinases
FoxM1: Forkhead box protein M1
Fra-1: Fos-related-antigen-1
Hh: Hedgehog
HCC: Hepatocellular carcinoma
HIV-1: Human immunodeficiency virus 1
HR: Homologous recombination
IFNs: Interferons
IκB: Inhibitor of NF-κB
IKK: IκB kinase
ISG15: Interferon-stimulated gene 15
IL: Interleukin
JAMMs: Jab1/MPN/MOV34 metalloenzymes
JNK: c-Jun N-terminal or stress-activated protein kinases
LncRNA: long non-coding RNA
MARK: microtubule-affinity regulating kinase
MAPK: mitogen-activated protein kinase
MAPKK: MAPK kinase
MAPKKK: MAPK kinase kinase
MEK2: mitogen-activated protein kinase kinase 2
MJDs: Machado-joseph disease proteases
MINDY: MIU-containing novel DUB family
mESCs: mouse embryonic stem cells
NDEA: nitrosodiethylamine
NEDD8: neural precursor cell expressed developmentally downregulated 8
NSCLC: non-small-cell lung cancer
NES: nuclear export sequence
NLSs: Nuclear localization signals
NoRC: nucleolar-remodeling complex
OTUs: Ovarian tumor proteases
PDAC: pancreatic ductal adenocarcinoma
PTCH: Patched
PKA: Protein kinase A
KCTD6: potassium channel tetramerization domain containing 6
PD-L1: programmed death ligand-1
PD-1: programmed death protein-1
RIP1: receptor interacting protein 1
RCC: renal cell carcinomas
Treg: Regulatory T
SMO: Smoothened
STING: stimulator of IFN genes
TCF/LEF: T-cell factor/lymphoid enhancer-binding factor
TcR: T cell receptor
TAK1: TGF-β activated kinase 1
Th1: T-helper type 1
Tat: transactivator of transcription
TNBC: triple negative breast cancer
TNFα: Tumor necrosis factor α
Ub: Ubiquitin
USP: ubiquitin-specific protease
USP21SV: USP21 short variant
UCHs: Ubiquitin carboxy-terminal hydrolases
XCI: X chromosome inactivation
ZUFSPs: Zn-finger and UFSP domain proteins.
